# Comparative Mitogenome Analysis of *Colletotrichum* Species Causing Anthracnose of Rubber Trees Unveils Distinct Species Complex-Specific Evolution Trajectories Within the Genus

**DOI:** 10.3390/jof11090679

**Published:** 2025-09-16

**Authors:** Yehao Wu, Fan Zhou, Qingqin Chen, Lijuan He, Yining Zang, Zirui Wang, Chunhua Lin, Weiguo Miao, Zhigang Li

**Affiliations:** 1 Sanya Institute of Breeding and Multiplication/School of Tropical Agriculture and Forestry, Hainan University, Haikou 570228, China; 2 Danzhou Invasive Species Observation and Research Station of Hainan Province, Hainan University, Danzhou 571799, China

**Keywords:** *Colletotrichum*, rubber tree, species complex, mitogenome, PCG arrangement, intron expansion/contraction

## Abstract

*Colletotrichum* spp. are the causative agents of anthracnose of rubber trees, one of the most destructive diseases, resulting in substantial economic losses. To investigate the evolutionary characteristics of these pathogenic species, we first assembled the complete mitogenomes of four dominant pathogens, i.e., *C. siamense*, *C. fructicola*, *C. wanningense* and *C. bannaense*. Comparative analyses revealed that variations in their mitogenome size were primarily driven by intron expansion and expansion/contraction within the *cox1*, *cob* and *nad* genes. Moreover, we observed the strong conservation of gene content, mitochondrial DNA copy number, gene order and intron features within species complexes, but a clear divergence between them. Notably, further studies indicated that patterns such as genomic organization, selective pressures and codon usage were consistent across the genus, suggesting that *Colletotrichum* species complexes had followed distinct evolutionary trajectories, particularly in the arrangement of protein-coding genes. Therefore, this study systematically characterized the mitogenomes of the four major *Colletotrichum* species associated with rubber tree anthracnose and provided novel insights into the broad evolutionary mechanisms shaping *Colletotrichum* species complexes.

## 1. Introduction

The rubber tree (*Hevea brasiliensis*) serves as the principal source of natural rubber in tropical regions and underpins a globally significant plantation industry [1]. Rubber production is increasingly threatened by foliar diseases that compromise tree health and latex yield, among which anthracnose is particularly damaging [2]. Anthracnose of rubber trees is caused by a complex assemblage of *Colletotrichum* species and leads to extensive necrosis, premature defoliation and substantial reductions in rubber yield [3]. Notably, surveys have identified more than ten distinct *Colletotrichum* taxa (in five species complexes) isolated from leaf disease lesions on *H. brasiliensis* in China, suggesting unexpectedly high species diversity [2,3]. However, the current research on these pathogenic species remains relatively limited. Up to now, only a few whole-genome sequences of *Colletotrichum* species that cause rubber tree anthracnose have been reported [4].

Among these *Colletotrichum* species, *C. siamense*, *C. fructicola*, *C. wanningense* (syn. *C. australisinense*) and *C. bannaense* have been investigated as the dominant pathogens that cause rubber tree anthracnose in China [3,4,5]. *C. siamense* and *C. fructicola* belong to the *C. gloeosporioides* species complex and *C. wanningense* and *C. bannaense* are members of the *C. acutatum* species complex. The four *Colletotrichum* species exhibit distinct lesion phenotypes, i.e., *C. siamense* and *C. fructicola* produce rapidly expanding brown to grayish lesions with unclear edges and *C. wanningense* forms large necrotic lesions with water-soaked edges, whereas *C. bannaense* causes small, shallow and weakly spreading lesions with a low pathogenicity. Additionally, these species exhibit a clear divergence in terms of their colony morphology, thermal adaptation and pathogenic potential [3,4,6,7].

In fungi, mitochondria serve as essential organelles that act as platforms for efficient energy metabolism and hubs for biosynthetic processes, and they play diverse roles in growth, environmental adaptation, aging, host–pathogen interactions and drug resistance [8,9,10,11,12]. Fungal mitogenomes evolve and organize autonomously, showing significant divergence from nuclear genomes. Fungal mitogenomes contain 15 typical protein-coding genes (PCGs) involved in the electron transport chain and oxidative phosphorylation, including those for Complex I (*nad1–nad6* and *nad4L*), Complex III (*cob*), Complex IV (*cox1–cox3*) and Complex V (*atp6*, *atp8*, *atp9*), as well as the gene encoding the ribosomal small subunit protein (*rps3*). In addition, fungal mitogenomes likely include non-coding RNA genes related to translation (such as *rnl*, *rns* and various tRNAs), the RNaseP gene (*rnpB*) and various intronic open reading frames (ORFs) [13]. The structures of mitogenomes vary widely; for example, they differ in their overall genome size, the number of introns and the gene order. Hitherto, variations in fungal mitogenomes have proven useful as molecular markers for reflecting phylogenetic relationships and population genetics [14]. Furthermore, the mitogenomes of fungal pathogens may be associated with their adaptation to host organisms [15,16]. However, to date, there have been no reports on the mitogenomes of *Colletotrichum* species that cause rubber tree anthracnose.

Therefore, in this study, we de novo assembled and analyzed the complete mitogenomes of the four major *Colletotrichum* species responsible for rubber tree anthracnose, i.e., *C. siamense* and *C. fructicola* (the *C. gloeosporioides* species complex) and *C. wanningense* and *C. bannaense* (the *C. acutatum* species complex). The genome structure, gene arrangement, intron content, mitochondrial DNA (mtDNA) copy number, codon usage and gene selective pressure were comprehensively analyzed, suggesting highly conserved characteristics within *Colletotrichum* species complexes, but significant variations between species complexes. A subsequent integrative analysis with an additional 30 *Colletotrichum* mitogenomes (comprising 19 species across 6 species complexes) revealed distinct species-complex specific evolution trajectories in the *Colletotrichum* genus, especially the PCG arrangement and intron expansion/contraction. Our study elucidated the mitogenomic variation among the *Colletotrichum* species that cause anthracnose of rubber trees and offers an evolutionary perspective into the specific evolutionary adaptation of *Colletotrichum* species complexes.

## 2. Materials and Methods

### 2.1. Single-Spore Isolation, DNA Extraction and Sequencing

*Colletotrichum* strains were isolated from anthracnose lesions on rubber tree (*Hevea brasiliensis*) leaves collected in Hainan Province, P. R. China. The infected tissues were surface-sterilized using 75% ethanol and rinsed with sterile distilled water before being cultured on potato dextrose agar (PDA) medium. Fungal isolates were purified using single-spore isolation under a stereomicroscope. The cultures were incubated at 28 °C in the dark for 7–10 days, and mycelia were harvested for DNA extraction. Genomic DNA was extracted using the cetyltrimethylammonium bromide (CTAB) method and subsequently purified using a T3010 genomic DNA purification kit (Tiangen Biotech, Beijing, China) according to the manufacturer’s instructions. The DNA integrity was verified using standard 0.75% agarose gel electrophoresis. The DNA concentration was quantified using a Qubit 3.0 fluorometer (Thermo Fisher Scientific, Waltham, MA, USA). Qualified genomic DNA (7 μg) was sheared to an average fragment size of 15–20 kb using the Megaruptor 3 system (Diagenode, B06010003) and purified with 1× AMPure PB beads. The sheared DNA was eluted in 47 μL of Low TE buffer and subjected to qubit quantification. SMRTbell^®^ libraries were constructed using the SMRTbell^®^ prep kit 3.0 (PacBio, Menlo Park, CA, USA), following the manufacturer’s protocol, including DNA damage repair, end-repair, adapter ligation and exonuclease treatment. A size selection of DNA fragments (10–50 kb) was performed using the PippinHT system (Sage Science, Beverly, MA, USA) and the fragment distribution was assessed on a Femto Pulse system (Agilent, Santa Clara, CA, USA). Primer annealing and polymerase binding were carried out according to the PacBio Revio sequencing protocol. SMRT sequencing was performed on the PacBio Revio™ system using the Revio™ sequencing plate (PacBio, 102-587-400), polymerase kit (PacBio, 102-739-100) and SMRT Cell tray (PacBio, 102-202-200) with a 24-h run time [17].

### 2.2. Mitogenome Assembly

Mitogenome assembly was performed using a custom pipeline based on the modified Mitochondrial Long-read Iterative Assembly (MLIA) strategy [17,18]. The assembly process involved the following steps: (1) HiFi reads were initially mapped to a reference mitogenome using minimap2 v2.30 with the -x map-hifi parameter [19,20]; (2) mapped reads shorter than 1000 bp or with aligned lengths less than 800 bp were filtered using seqtk v1.3 (https://github.com/lh3/seqtk, accessed on 15 July 2025); and (3) filtered reads were assembled de novo using Canu v2.1.1 with the parameters optimized for HiFi input (-pacbio-hifi) [21,22]. The resulting contigs were then used as the reference for the next round of read recruitment and reassembly. This iterative process continued until the number of recruited mitochondrial reads reached a stable plateau and a complete circular contig representing the mitogenome was obtained.

### 2.3. mtDNA Copy Number Detection

The mycelia DNA was extracted from fungal mycelia using the E.Z.N.A.^®^ Fungal DNA Kit (Omega Bio-Tek, D3390-10) according to the manufacturer’s instructions. Relative mtDNA copy number was determined by qPCR, with the nuclear single-copy gene *RPB1* used as the reference and the mitochondrial gene *NAD4* as the target [23,24]. Primers for *RPB1* were 5′-ATGAAGCAGGCTCCCGT-3′ (forward) and 5′-GGCTCCTCCTTCTTCTTGAC-3′ (reverse) and primers for *NAD4* were 5′-GCTCACGGATTTGTATCTAGTGG-3′ (forward) and 5′-GTTAAAGGAGCTCCACAATTACCT-3′ (reverse). The Ct difference between *RPB1* and *NAD4* was used to estimate the relative mtDNA copy number.

### 2.4. Genome Annotation

The assembled mitogenomes were annotated using MFannot with the genetic code 4 (Mold, Protozoan and Coelenterate Mitochondrial; Mycoplasma/Spiroplasma) [25]. The tRNA genes were predicted using both MFannot and MITOS2 for cross-validation [25,26]. Group I and Group II introns were identified and classified using RNAweasel [27,28]. Intronic ORFs were predicted using ORFfinder v0.4.3 [29] and the transeq tool (with genetic code 4) in the EMBOSS v6.6.0 package [30]. Functional domains within intronic ORFs, including the reverse transcriptases (RTs) and homing endonucleases (HEs) of the LAGLIDADG or GIY-YIG families, were identified using the NCBI Conserved Domain Database (E-value < 1 ×10^−^^3^) [31]. Short repeats and tandem repeats were identified using REPuter [32] and Tandem Repeat Finder v4.09 [33], respectively, under default settings. Whole-genome repetitive sequences were additionally annotated through BLASTN self-alignment (E-value < 1 ×10^−10^). The circular mitogenome maps displaying the arrangement and orientation of annotated features were generated using Circos v0.69-6 [34].

### 2.5. Synteny Analysis

The nucleotide-level homology was identified using BLASTN (E-value < 1 ×10^−5^) [35]. The resulting BLAST outputs were processed and visualized using a genome collinearity mapping tool called RectChr v1.1 (https://github.com/BGI-shenzhen/RectChr, accessed on 15 July 2025). Gene annotations were integrated to anchor syntenic blocks and highlight conserved PCGs, intron positions and genome rearrangements (e.g., inversions and translocations).

### 2.6. Phylogenetic Analysis Based on PCGs

Multiple assembled mitogenome sequences were collected from the NCBI nucleotide database. Genome sequences exhibiting incomplete assemblies, anomalously large or small sizes, or structural inconsistencies (e.g., duplicated core genes or truncated annotations) were excluded from the dataset to ensure phylogenetic reliability.

A total of 15 conserved PCGs (i.e., nad1-nad6, nad4L, cox1-cox3, cob, atp6, atp8, atp9 and rps3) were extracted. Each gene was aligned independently using MUSCLE v5.1 [36] using the codon alignment mode. The resulting alignments were concatenated into a supermatrix for phylogenetic inference. A maximum likelihood (ML) phylogenetic tree was constructed using IQ-TREE v2.2.2 [37] with the best-fit substitution model selected automatically. The branch support was assessed using 1000 bootstrap replicates under the standard bootstrap approach. The final tree was visualized and annotated using the Interactive Tree of Life (iTOL) v6 [38].

### 2.7. Composition, Codon Usage and Substitution Rate Analyses

The base composition of each mitogenome was calculated using SeqKit v2.2.0 [39]. The GC-skew and AT-skew were computed manually with the following formulas: GC-skew = (G − C)/(G + C) and AT-skew = (A − T)/(A + T). The codon usage statistics, including the codon frequencies and relative synonymous codon usage (RSCU) values, were obtained using BCAWT [40] and the Python v3.12 Codon Adaptation Index (CAI) module (https://pypi.org/project/CAI/, accessed on 15 July 2025). A total of 60 codons (excluding stop codons and the unique codon TGG for tryptophan) were assessed. The clustering of codon usage patterns was achieved using CIMminer [41], applying Euclidean distance and average linkage methods. To evaluate the codon usage patterns in *Colletotrichum* mitogenomes, RSCU values of 15 core PCGs were calculated and compared across 34 representative taxa. The non-synonymous substitution rates (dN), synonymous substitution rates (dS) and their ratios (dN/dS) were calculated using the yn00 program in the PAML v4.10.3 package [42,43], based on the method by Yang & Nielsen (2000). PCG orthologs in Colletotrichum species complexes with >2 species were extracted for the dN/dS calculation [44]. Codon-based sequence alignments in the dN/dS calculation steps were performed using MAFFT v7.505 [45] under genetic code 4.

### 2.8. Program and Data Availability

The MLIA pipeline is available on GitHub (https://github.com/lirepo/MLIA, accessed on 15 July 2025). The de novo assembled mitogenomes in this study are available in the GenBank database, i.e., *C. siamense* (CP150621.1), *C. fructicola* (CP150825.1), *C. wanningense* (PV763180.1) and *C. bannaense* (PV763181.1).

## 3. Results

### 3.1. De Novo Assembly and Structural Annotation

The gapless circular mitogenomes of the four major *Colletotrichum* species (i.e., *C. siamense*, *C. fructicola*, *C. wanningense* and *C. bannaense*) that cause anthracnose of rubber trees in China were assembled in this study. Among them, the complete mitogenomes of *C. wanningense* and *C. bannaense* were assembled for the first time in this study.

**Figure 1:**
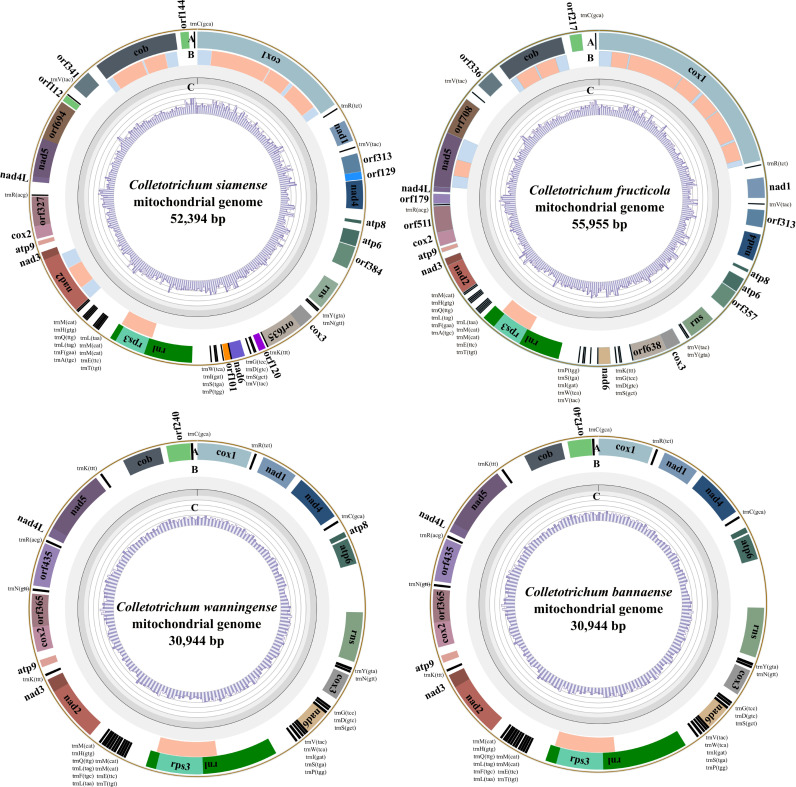
Genomic features of the four de novo assembled mitogenomes of *Colletotrichum* species. The circular maps represent the mitogenomics of *C. siamense*, *C. fructicola*, *C. wanningense* and *C. bannaense*. (A) Annotated genes such as protein-coding genes, rRNA genes and tRNA genes. (B) Labeled gene structures with introns. Blue and orange tracks represent the exon and intron regions, respectively. (C) GC content distribution along each mitogenome.

The complete mitogenomes of *C. siamense* and *C. fructicola*, belonging to the *C. gloeosporioides* species complex, were 52,394 bp and 55,955 bp in size, respectively. The mitogenomes of the two species had overall GC contents of 34.52% and 33.98%, respectively. In contrast, the mitogenomes of *C. wanningense* and *C. bannaense*, which are *C. acutatum* species complex members, were 30,949 bp and 30,944 bp in size, respectively. Both mitogenomes exhibited the same GC content, 30.59% (Figure 1; Table 1).

The mitochondrial core PCGs were conserved among the four *Colletotrichum* species. The *C. siamense* and *C. fructicola* mitogenomes contained 27 tRNA genes, while *C. wanningense* and *C. bannaense* had 28 tRNA genes. Compared to the slight difference in tRNA genes, the homing endonuclease ORFs belonging to the LAGLIDADG and GIY-YIG families were observed in *C. siamense* and *C. fructicola*, but absent in *C. bannaense* and *C. wanningense*, indicating their specific acquisition and maintenance of genetic elements.

**Figure 2:**
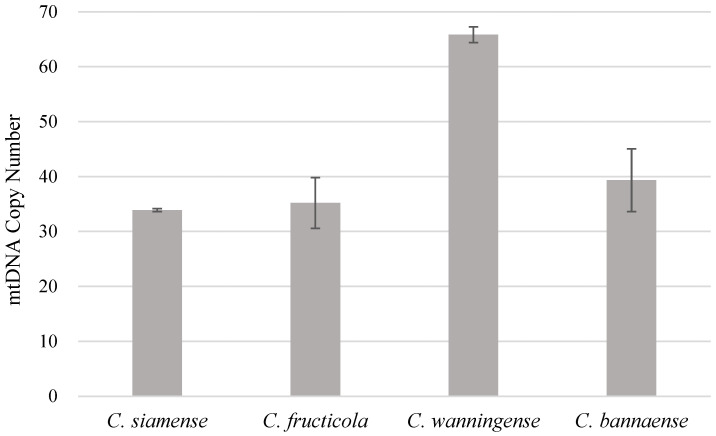
Estimated relative mtDNA copy numbers of the four *Colletotrichum* species. Relative mtDNA copy number of four *Colletotrichum* species estimated by qPCR, using nuclear single-copy gene *RPB1* as the reference and mitochondrial gene *NAD4* as the target.

The mtDNA copy numbers of four *Colletotrichum* species were estimated by qPCR (Figure 2). Notable differences were observed among species: *C. siamense* and *C. fructicola* exhibited lower copy numbers (~35), whereas *C. bannaense* (~40) and *C. wanningense* (~65) showed higher values. Overall, the *C. acutatum* complex species had higher mtDNA copy numbers than the *C. gloeosporioides* complex species (*t*-test, *p* = 0.03).

### 3.2. Comparative Synteny Analysis Revealed Intron-Driven Genome Expansion

**Figure 3:**
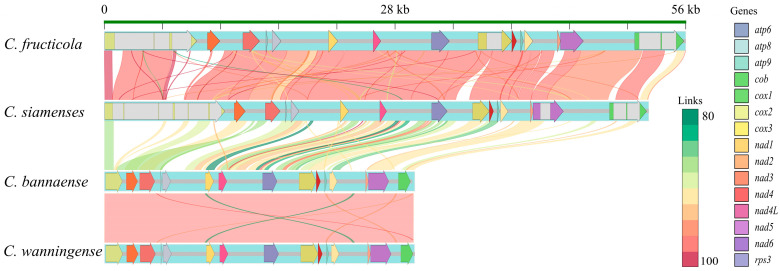
Synteny comparison of mitogenomes among the four *Colletotrichum* species. Thick tracks denote protein-coding gene regions, with arrows indicating the transcriptional direction. The colors represent exon regions in different genes and the gray regions indicate intron regions. The ribbon connects homologous regions identified by BLASTN (E-value < 1 × 10^−5^), with the color intensity reflecting the sequence identity.

The four assembled mitogenomes displayed a conserved PCG content and order (Figure 3). The orientation of the PCGs remained consistent as well. However, the mitogenome sizes differed markedly. *C. siamense* and *C. fructicola* exhibited significantly larger mitogenomes (52,394 bp and 55,955 bp, respectively) than *C. bannaense* and *C. wanningense* (~30,944 bp). This size difference was primarily due to variations in the intronic regions, e.g., *cox1*, *cob*, *nad2* and *nad4* in *C. fructicola* and *C. siamense*. In contrast, *C. bannaense* and *C. wanningense* contained only one intron in their mitogenomes, resulting in a more compact genome structure. The mitogenomic regions were largely syntenic, as indicated by extensive BLASTN-derived sequence similarities.

A detailed analysis of the pairwise BLASTN alignment revealed relatively minor structural differences. *C. siamense* and *C. fructicola* exhibited extensive regions of a high sequence identity (≥95%) across nearly all coding regions, frequently spanning thousands of continuous base pairs without gaps. Several small insertions were detected in the *C. fructicola* mitogenome, notably within the 8.8 kb–16 kb and 24 kb–45 kb regions, corresponding to segments within or adjacent to the cox1, cob and nad genes.

The alignment of *C. wanningense* and *C. bannaense* showed a nearly perfect sequence identity across the entire mitogenome. Specifically, they exhibited a continuous 30,828 bp alignment with 99.93% identity and without gaps, highlighting an extremely high degree of structural conservation. No minor insertions or expansions were observed in the two species, suggesting a more conserved genomic structure in these two species compared to the two species that belong to the *C. gloeosporioides* species complex.

### 3.3. Structural Diversity of Colletotrichum Mitogenomics

To further systematically explore the evolutionary characteristics of the four *Colletotrichum* species, we collected 30 previously assembled mitogenomes of *Colletotrichum* species from the GenBank database (Table 2). The genome sizes of these mitogenomes were from ~29 kb (*C. scovillei*) to ~60 kb (*C. gloeosporioides*) and the GC contents were between 29.6% and 34.6%. Finally, a total of 34 mitogenomes, including the four newly assembled mitogenomes in our study, were subsequently analyzed.

**Figure 4:**
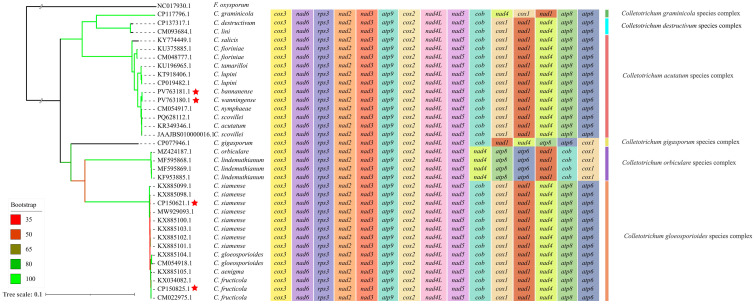
Maximum likelihood phylogeny based on 15 PCGs from 34 *Colletotrichum* species. The tree was constructed using 15 concatenated PCGs (i.e., *cox1–3*, *cob*, *atp6*, *atp8*, *atp9*, *nad1–6*, *nad4L* and *rps3*). *Fusarium oxysporum* (NC017930.1) was included as an outgroup. The bootstrap values are indicated by the branch color. The *Colletotrichum* mitogenomes that were newly assembled in this study are marked by red asterisks. Species from six *Colletotrichum* species complexes are colored and grouped by vertical bars.

A comparative analysis of conserved PCG arrangements revealed the distinct structural differentiation of mitogenomes across *Colletotrichum* species complexes. The gene order was highly conserved within species complexes, while significant variations were observed between species complexes (Figure 4). *C. siamense*, *C. fructicola* and *C. gloeosporioides* (*C. gloeosporioides* species complex) shared an identical arrangement of 15 PCGs. Analogously, the examined *C. fioriniae*, *C. tamarilloi*, *C. acutatum*, *C. lupini* and *C. scovillei* (*C. acutatum* species complex) exhibited a fully conserved gene order.

*C. gigasporum* (*C. gigasporum* species complex) displayed a rearranged gene order, with the *cox1* gene relocated to the terminal region of the sequence, clearly differing from the conserved order observed in the *C. acutatum* species complex and the *C. gloeosporioides* species complex. Likewise, *C. orbiculare* and *C. lindemuthianum* (*C. orbiculare* species complex) exhibited a unique gene order pattern, with *cob*, *nad1*, *nad4*, *atp8* and *atp6* arranged differently from those in the other complexes. The gene order within the *C. destructivum* species complex and the *C. graminicola* species complex remained highly consistent, but they exhibited distinct gene arrangements for *nad4* and *cox1*. These findings indicate that the *Colletotrichum* genus exhibits species complex-specific gene-arrangement patterns. Thus, the mitochondrial gene arrangement serves as an informative evolutionary trait and supports the delineation of species complexes as fundamental phylogenetic units within the genus.

### 3.4. Gene Selective Pressure Analysis

**Figure 5:**
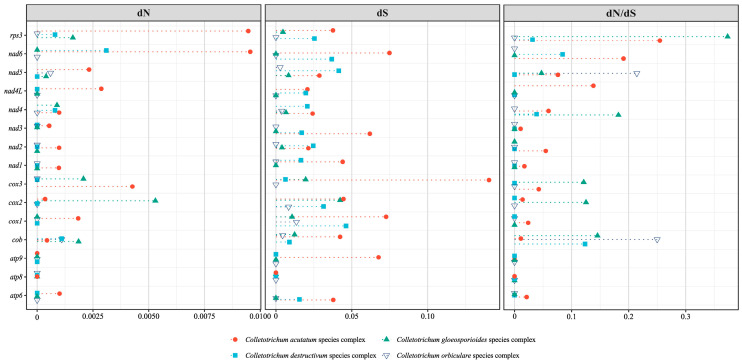
Substitution rate analysis for 15 PCGs in *Colletotrichum* species complexes. Point diagram showing the dN, dS and dN/dS values of each gene in different *Colletotrichum* species complexes. The horizontal axis represents the dN, dS and dN/dS values.

In general, the dN/dS values were predominantly below 0.1 (Figure 5). Most PCGs exhibited low dN and dS values across nearly all the species complexes. For example, *atp6*, *cox1*, *cox2*, *cob* and *nad3* showed low dN values. *Atp8* and *atp9* had dN values of zero in most complexes. In some genes, the dS values were equal to zero, making dN/dS undefined, and thus, not reported.

In the *C. acutatum* species complex, the dN/dS ratios were 0.021 for *atp6*, 0.024 for cox1, 0.017 for *nad1* and 0.014 for *cox2*. Higher values were observed for *rps3* (0.255), *nad6* (0.191) and *nad4L* (0.138). Other genes, such as *nad5* (0.076), *nad4* (0.060), *nad2* (0.055) and *cox3* (0.042), also showed non-zero values. In the *C. destructivum* species complex, most genes had dN values of zero. *Cob* had a dN of 0.0011 (dN/dS = 0.124), *nad6* had a dN of 0.0031 (dN/dS = 0.084) and *nad4* had a dN of 0.0008 (dN/dS = 0.039). *Rps3* showed a dN/dS of 0.031. In the *C. orbiculare* species complex, most genes had dN values of zero. *Cob* and *nad5* showed dN/dS values of 0.250 and 0.214, respectively. In the *C. gloeosporioides* species complex, the dN/dS ratios were observed across the genes as follows: 0.373 (*rps3*), 0.182 (*nad4*), 0.145 (*cob*), 0.125 (*cox2*) and 0.121 (*cox3*).

### 3.5. Codon Usage Patterns Reflect Translational Stability Within Species Complexes

**Figure 6:**
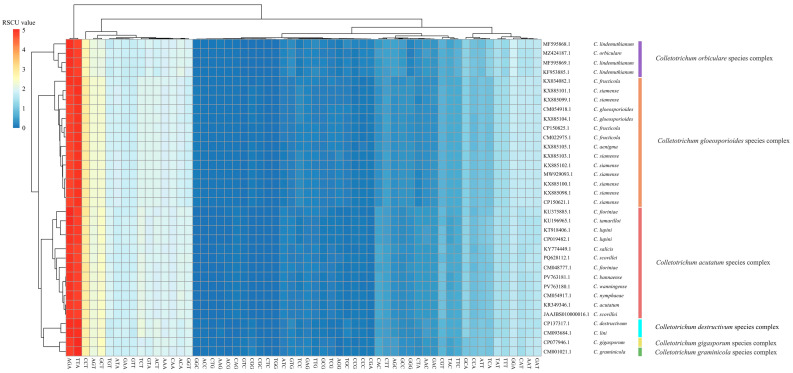
Heatmap of RSCU across 34 *Colletotrichum* mitogenomes. RSCU values for 59 non-stop codons are shown, with colors ranging from blue (low usage) to red (high usage). Hierarchical clustering based on the Euclidean distance revealed the codon usage similarity within and across species complexes.

Overall, a strong bias toward A/T-ending codons was observed (Figure 6). For example, TTA (Leu) and AGA (Arg) exhibited the highest average RSCU values of 4.77 and 4.74, respectively, followed by CCT (Pro), GCT (Ala) and AGT (Ser). These codons showed the most pronounced usage preference in the *Colletotrichum* genus. In contrast, codons such as CGC (Arg), TGG (Trp) and CCG (Pro) had consistently low RSCU values, some of which were close to zero. These codons were rarely used or nearly completely avoided in *Colletotrichum* mitogenomes.

Hierarchical clustering based on the RSCU values revealed clear cluster patterns that corresponded closely with species complex boundaries (Figure 6). For instance, the *C. gloeosporioides* species complex, *C. acutatum* species complex and *C. destructivum* species complex formed distinct clusters, indicating that the codon usage patterns are highly similar within species complexes. Additionally, the *C. gloeosporioides* species complex, *C. truncatum* species complex and *C. gigasporum* species complex formed well-separated branches, further supporting intra-group codon usage coherence.

## 4. Discussion

This study de novo assembled the mitogenomes of four major *Colletotrichum* species that cause anthracnose of rubber trees in China, i.e., *C. siamense*, *C. fructicola*, *C. wanningense* and *C. bannaense*. The mitogenome sizes of *C. siamense* and *C. fructicola* (*C. gloeosporioides* species complex) were ~52–56 kb, significantly larger than the ~30 kb of *C. wanningense* and *C. bannaense* (*C. acutatum* species complex). Intron expansion was found to be the primary driver of genome size variation. Species in the *C. gloeosporioides* species complex, for example, contained ten introns, whereas members of the *C. acutatum* species complex carried only one intron. These intronic regions in genes such as *cox1*, *cob* and *nad* play a central role in shaping mitogenome sizes. A systematic analysis of the four mitogenomes revealed highly conserved features within species complexes, particularly in the structure and arrangement of core PCGs, whereas substantial structural differences were observed between species complexes. Our analysis revealed clear interspecific differences in relative mtDNA copy number among *Colletotrichum*. Species of the *C. acutatum* complex exhibited higher copy numbers than those of the *C. gloeosporioides* complex. This study provides the first evidence of mtDNA copy number variation between *Colletotrichum* species complexes, suggesting that such variation may contribute to phenotypic divergence. Further studies with broader sampling are needed to confirm these observations.

To establish corroboration for our hypothesis of distinct species-complex specific evolution trajectories within *Colletotrichum*, this study subsequently provided a more comprehensive analysis of mitochondrial diversity by including a broader range of taxonomic species across multiple *Colletotrichum* species complexes. A total of 34 complete mitogenomes of *Colletotrichum* species, with genome sizes ranging from ~29 kb (*C. scovillei*) to ~60 kb (*C. gloeosporioides*) and GC contents between 29.6% and 34.6%, were systematically analyzed.

Intron invasion was confirmed as being a major contributor to mitogenome expansion in *Colletotrichum* species, consistent with previous reports in some filamentous fungi [46]. Earlier studies have suggested that self-splicing introns, particularly types I and II, often spread through homing endonuclease genes (HEGs) [14]. Our genome-wide detection of LAGLIDADG and GIY-YIG ORFs in *Colletotrichum* species (e.g., our newly assembled *C. siamense* and *C. fructicola* mitogenomes) supports this mechanism. The species-complex specific intron patterns imply that HEG mobility may be influenced by the species ecology, host range and life cycle [47].

Notably, our study first uncovered that the mitochondrial gene order is conserved within *Colletotrichum* species complexes, but structurally rearranged between different complexes. The concept of a species complex was initially introduced to address cryptic species that are morphologically indistinguishable, but genetically divergent at the molecular level [48,49]. Interestingly, our results show that the mitochondrial gene order remains completely conserved among such species within the same complex. Conversely, species in different species complexes (e.g., *C. gigasporum* and *C. orbiculare*) exhibited distinct mitochondrial gene arrangements. In fungi, such structural rearrangements are associated with altered gene regulation, reduced mitochondrial compatibility and even reproductive barriers [50]. Structural stability within species complexes may thus reflect the conservation of bioenergetic functions, particularly among taxa that share similar ecological niches.

Given the essential role of mitochondria in cellular respiration, the integrity of the mitogenome architecture was likely under intense selective pressure [51]. A selection pressure analysis of *Colletotrichum* species complexes revealed that most genes were under strong purifying selection, indicating strict functional constraints. The *C. gloeosporioides* species complex is known for its wide host range and ecological plasticity [52]. Although most PCGs in the *C. gloeosporioides* species complex were also under strong purifying selection, the dN/dS ratios of several genes (e.g., *rps3*, *cox3* and *nad4*) showed elevated dN/dS ratios, with *rps3* reaching the highest value of 0.373, suggesting potential adaptive divergence. *Rps3* had the highest dN/dS value in all species complexes for its location within the *rnl* intron and its evolution may have been influenced by non-adaptive forces such as intron mobility and splicing regulation [53,54]. *Rps3* could emerge as a promising molecular marker due to its high sequence variability, although further validation is needed to confirm its robustness across taxa. While intron mobility could be an important contributor to the elevated dN/dS values in *rps3* and other genes, alternative explanations should also be considered. For example, adaptive modifications in mitochondrial proteins may alter energy metabolism and facilitate infection across different hosts. Moreover, the relatively high dN/dS values detected might be partly attributable to the small sample sizes. These possibilities warrant further investigation with expanded datasets in future studies.

The codon usage patterns were highly consistent across *Colletotrichum* species. A clear codon preference was observed for A/T-ending codons, such as TTA (Leu) and AGA (Arg). The observed codon bias toward A/T-ending codons was putatively due to two major forces: the high A/T content in the mitogenome and a minimized tRNA repertoire favoring those codons [55]. These conserved codon usage patterns clustered strongly within species complexes, indicating that translational evolution may offer an auxiliary classification signal.

Our study revealed that a high level of conservation within *Colletotrichum* complexes, e.g., gene structure, gene arrangement, selection pressure and codon usage, indicates evolutionary stability. For instance, in the *C. acutatum* species complex, *C. bannaense* and *C. wanningense* had near-identical sequences and *C. acutatum*, *C. fioriniae* and *C. tamarilloi* exhibited minimal mitochondrial variation. This observation also suggests that *Colletotrichum* mitogenomes may offer a limited resolution for species delimitation in species complexes that rely on only a single or a few loci, e.g., *rps3* [13].

In summary, we newly assembled the mitogenomes of four major *Colletotrichum* species that cause rubber tree anthracnose. Our comprehensive mitogenomic analysis revealed the remarkable conservation of the mitochondrial PCG arrangement within *Colletotrichum* species complexes, but these arrangements differed between distinct species complexes. Concurrently, substantial structural divergences were observed, driven primarily by intronic expansion/contraction and mobile genetic elements. Furthermore, selective pressures and codon usage patterns exhibit distinct evolutionary dynamics at the species complex level. Therefore, the findings of this study systematically present the characteristics of the four major *Colletotrichum* species responsible for anthracnose of rubber trees and offer novel insights into the broad evolutionary mechanisms underlying *Colletotrichum* species complexes.

## Figures and Tables

**Table 1:** Assembly statistics and genomic features of mitogenomes.

	*C. siamense*	*C. fructicola*	*C. wanningense*	*C. bannaense*
GenBank Accession	CP150621.1	CP150825.1	PV763180.1	PV763181.1
Total size (bp)	52,394	55,955	30,949	30,944
Intergenic regions size (bp)	12,555	13,952	9649	9644
Intronic regions size (bp)	5609	8383	1709	1709
Overall GC (%)	34.52	33.98	30.59	30.59
Core PCGs size (bp)	27,267	30,050	16,237	16,237
Intergenic regions GC (%)	41.35	41.97	30.95	30.59
GC-skew(G − C)/(G + C)	0.07	0.07	0.11	0.11
AT-skew(A − T)/(A + T)	−0.01	0	−0.03	−0.03
Repetitive DNA regions (bp)	1579	1215	208	208
Repetitive DNA (%)	3.01	2.17	0.67	0.67
tRNA	27	27	28	28
Introns	7	10	1	1
Intronic ORFs	1	1	1	1
GIY-YIG ORFs	1	1	0	0
LAGLIDADG ORFs	4	5	0	0

**Table 2:** Mitogenomic characteristics of *Colletotrichum* species in the GenBank database.

GenBank Accession	Species	Species Complex	Genome Length (bp)	GC Content (%)	tRNA Count	rRNA Count	Intron Count
CM093684.1	*C. lini*	*C. destructivum* species complex	39,089	29.62	28	2	3
CP137317.1	*C. destructivum*	*C. destructivum* species complex	34,391	29.72	28	2	2
CM001021.1	*C. graminicola*	*C. graminicola* species complex	39,649	29.89	25	2	2
KY774449.1	*C. salicis*	*C. acutatum* species complex	33,950	30.44	28	2	1
KU375885.1	*C. fioriniae*	*C. acutatum* species complex	30,020	30.04	28	2	1
CM048777.1	*C. fioriniae*	*C. acutatum* species complex	30,009	30.10	29	2	1
KU196965.1	*C. tamarilloi*	*C. acutatum* species complex	30,824	30.50	28	2	1
KT918406.1	*C. lupini*	*C. acutatum* species complex	36,554	29.91	29	2	1
CP019482.1	*C. lupini*	*C. acutatum* species complex	36,554	29.91	29	2	1
PV763180 *	*C. wanningense*	*C. acutatum* species complex	30,949	30.59	28	2	1
PV763181 *	*C. bannaense*	*C. acutatum* species complex	30,944	30.59	28	2	1
CM054917.1	*C. nymphaeae*	*C. acutatum* species complex	30,928	30.54	28	2	1
KR349346.1	*C. acutatum*	*C. acutatum* species complex	30,892	30.51	28	2	1
PQ628112.1	*C. scovillei*	*C. acutatum* species complex	29,517	30.72	21	2	1
JAAJBS010000016.1	*C. scovillei*	*C. acutatum* species complex	30,952	30.51	28	2	1
CP077946.1	*C. gigasporum*	*C. gigasporum* species complex	58,879	31.97	29	2	11
MZ424187.1	*C. orbiculare*	*C. orbiculare* species complex	36,318	31.07	28	2	3
MF595869.1	*C. lindemuthianum*	*C. orbiculare* species complex	37,446	30.86	28	2	4
MF595868.1	*C. lindemuthianum*	*C. orbiculare* species complex	37,440	30.85	28	2	4
KF953885.1	*C. lindemuthianum*	*C. orbiculare* species complex	36,957	30.88	28	2	4
KX885098.1	*C. siamense*	*C. gloeosporioides* species complex	54,679	34.25	27	2	9
KX885099.1	*C. siamense*	*C. gloeosporioides* species complex	54,658	34.10	27	2	9
KX885102.1	*C. siamense*	*C. gloeosporioides* species complex	54,645	34.29	27	2	8
KX885103.1	*C. siamense*	*C. gloeosporioides* species complex	53,317	34.39	27	2	7
MW929093.1	*C. siamense*	*C. gloeosporioides* species complex	52,710	34.45	27	2	7
KX885100.1	*C. siamense*	*C. gloeosporioides* species complex	52,671	34.40	27	2	7
CP150621.1 *	*C. siamense*	*C. gloeosporioides* species complex	52,394	34.52	27	2	7
KX885101.1	*C. siamense*	*C. gloeosporioides* species complex	58,666	33.84	27	2	11
CM054918.1	*C. gloeosporioides*	*C. gloeosporioides* species complex	59,695	34.13	27	2	11
KX885105.1	*C. aenigma*	*C. gloeosporioides* species complex	57,252	34.28	27	2	10
KX034082.1	*C. fructicola*	*C. gloeosporioides* species complex	56,051	34.04	27	2	10
CP150825.1 *	*C. fructicola*	*C. gloeosporioides* species complex	55,955	33.98	27	2	10
CM022975.1	*C. fructicola*	*C. gloeosporioides* species complex	55,177	34.00	27	2	10
KX885104.1	*C. gloeosporioides*	*C. gloeosporioides* species complex	55,169	34.55	27	2	9

The mitogenomes that were newly assembled in this study are marked by *.

## Data Availability

The de novo assembled mitogenomes in this study were available in the GenBank database, i.e., *C. siamense* (CP150621.1), *C. fructicola* (CP150825.1), *C. wanningense* (PV763180.1), and *C. bannaense* (PV763181.1).
